# Effects of the single nucleotide polymorphism at MDM2 309 on breast cancer patients with/without BRCA1/2 mutations

**DOI:** 10.1186/1471-2407-9-60

**Published:** 2009-02-18

**Authors:** Hovav Nechushtan, Tamar Hamburger, Susan Mendelson, Luna Kadouri, Nir Sharon, Eli Pikarsky, Tamar Peretz

**Affiliations:** 1 Department of Oncology, Hadassah Hebrew University Medical Center, Jerusalem 91120, Israel; 2 Department of Public Health, Hadassah Hebrew University Medical Center, Jerusalem 91120, Israel; 3 Department of Pathology, Hadassah Hebrew University Medical Center, Jerusalem 91120, Israel

## Abstract

**Background:**

A germ line single nucleotide polymorphism (SNP) in the first intron of the gene encoding MDM2 at position 309, an important modulator of p53, has been described. BRCA1/2 mutation have been associated with increased rates of breast cancers with mutated P53. It was shown that the presence of MDM2 309 SNP correlated with younger cancer onset age in individuals with a p53 mutations. The differential effects of this SNP were also linked to estrogen receptor activation. Here we report on our study of 453 Ashkenazi breast cancer patients of whom 180 were positive for the known Ashkenazi BRCA1/2 mutations

**Methods:**

DNA from breast cancer patients was obtained for analysis of one of the three common BRCA1/2 mutations and MDM2 SNP309. Data regarding cancer onset and death ages was obtained from our database and Statistical analysis was performed using the SPSS^® ^statistical package (SPCC Inc., Chicago, IL), and JMP^® ^software (SAS Institute, Cary, NC).

**Results:**

The percentage of MDM2 SNP309 in control and BRCA 1/2 population which is similar to that reported for other Jewish Ashkenazi populations at 52.2% for the heterozygotes and 25.0% for MDM2SNP309G/G and 22.8% for MDM2SNP309T/T.

There was not a statistical significant difference in median age of disease onset in the different MDM2 SNP309 subgroups of the BRCA1/2 carriers. When we further divided the group into under and above 51 years old ( presumed menopause age) in the BRCA1 positive subset we found that there were less patients of the MDM2SNP309 G/G versus the MDM2SNP309 T/T in the over 51 patient group (p = 0.049). This result has been obtained in a relatively small subgroup and is of borderline statistical significance. Interestingly, in the BRCA1/2 mutation carriers, we found a survival advantage for patients harboring the SNP309 G/G genotype (p = 0.0086) but not for the 272 patients not harbouring this mutations.

**Conclusion:**

MDM2SNP309G/G main effect on BRCA1/2 positive mutation carriers is linked to its effect on patients survival. Further research is needed in order to understand the reason for this difference.

## Background

MDM2 regulates p53 by targeting its destruction through the ubiquitin pathway and also by directly blocking p53 transcriptional activity[1]. A single nucleotide polymorphism (SNP) in the MDM2 promoter (SNP309) was identified [2]. Homozygotic SNP309 G/G carriers express higher levels of MDM2, which can subsequently attenuate the p53 pathway [2]. A significantly reduced age of onset for several p53 dependent cancers have been described in SNP309 G/G homozygous carriers including patients with Li-Fraumeni syndrome [2]. In contrast, studies have also shown that SNP309 G/G alone does not have an effect on the risk or the onset of various cancers, including familial breast cancer in which mutations for BRCA1 or BRCA2 have not been detected [3].

Two recent articles have studied this subject again with somewhat conflicting results. Yarden et al [4], studied BRCA1/2 mutation carriers. They divided this population into two – those under 51 years old (probably premenopausal), and those over 51 years old. In the under 51 years old they found a much larger fraction of patients harboring MDM2 SNP309G/G compared to the fraction of patients harbouring this SNP in the over 51 years old, supporting a role for this SNP as a modifier of cancer risk in BRCA1/2 mutation carriers. Wasielewski et al [5], described a younger cancer onset age for MDM2 SNP309 G/G carriers in a large heterogenous group of patients with familial breast cancer. However the MDM2 SNP309 G/G correlation with cancer onset age in their study seemed to be restricted to non mutant breast familial cancer cases and was not apparent in the BRCA1/2 mutation carriers. Bond, Levine and colleagues studied the effects of SNP309 G/G on breast cancer onset age in a group of Ashkenazi breast cancer patients who were not BRCA1/2 carriers of mutations. They noted a significantly earlier cancer onset in women with both a strongly positive estrogen receptor expression pattern and the G/G polymorphism at the MDM2 309 position [6]. Interestingly, in the subset without a strongly positive estrogen receptor expression pattern, there was a trend for disease onset at an older age in the SNP309 G/G carriers compared to the SNP309 T/T carriers(p = 0.1). Thus different results as to the effect of MDM2 309 SNP on breast cancer onset age in BRCA1/2 mutation carriers and in the non carrier control Ashkenazi group were obtained by different groups.

Several studies have found negative correlations between MDM2 tumor expression and prognosis in various cancers including breast, ovarian and brain [7-9]. Two studies have also noted a negative effect of MDM2 SNP309 G/G on survival of patients with metastatic gastric and renal cell cancer[10,11]. However in breast cancer a more complex picture emerges from a study by Mathoulin-Portie et al[12]. This group stained breast cancer tumor samples for p53, MDM2 and p21 (a p53 regulated gene)and analyzed survival of those patients following adjuvant anthracyclines containing treatments. In their study, the p53+/p21+/MDM2+ tumors were associated with a better outcome than the p53+/p21+/MDM2- tumors. These results imply that the relationship between MDM2 expression and survival is not simple and may depend on specific tumor characteristics and clinical circumstances.

Estrogen seems also to influence the magnitude of the MDM2 SNP309 G/G effects on MDM2 expression. Estrogen receptor which has a DNA binding site close to the Sp1 site which is directly influences by MDM2 SNP309 G/G. Bond, Levine and colleagues have described a significant correlation between MDM2 SNP309 G/G and the prevalence of diffuse large cell lymphoma and soft tissue sarcoma, in women under the age of 51 (persumably premenopausal) [6]. This findings support a role for estrogen in the MDM2 SNP309 G/G effects on carcinogenesis.

We have obtained DNA samples from a large group of breast cancer patients (nearly all Jewish Ashkenazi) for analysis of BRCA1/2 mutations and modifier genes. Here we describe the interactions we found out in this group between MDM2 SNP309 G/G, BRCA1/2 mutations and clinical findings including cancer onset age, survival, and the presence of other cancers.

## Methods

### Study population

DNA was from breast cancer patients obtained for analysis of their BRCA status and modifier genes (see Table 1 for specific mutations). These patient population does not contain all breast cancer patients presenting to our clinic but all of those who agreed for genetic testing. Thus this patient population presumably contains a relatively high percentage of breast cancer patients who had reasons to agree for genetic testings such as other cases of cancer in the family, younger cancer onset age etc. All participants signed an informed consent approved by the institutional ethics committee. In the Jewish Ashkenazi population there is an especially high prevalence of BRCA1/2 [13,14], which constitute a high percentage of our patient population. All of the patients had been diagnosed with breast cancer and analyzed for one of the common three Ashkenazi mutations in the BRCA genes which account for probably more than 95% of all Ashkenazi patients harboring BRCA mutations.

**Table 1:** BRCA status of 180 Ashkenazi breast cancer BRCA positive patients

Mutation	Frequency	Percent
BRCA1	111	61.6
BRCA2	68	37.7
BRCA1+2	1	0.6
**Total**	**180**	

### Genotyping

Genomic DNA was extracted according to standard protocols, and used as a template for the PCR reaction. BRCA mutations were analyzed by a combiniation of multiplex PCR with specific primers, and restriction site analysis as described in detail by Abeilovich and colleagues [15]. MDM2 was analyzed for SNPs using either bidirectional sequencing of a fragment of DNA amplified from the MDM2 human promoter (primer 1: CGGGAGTTCAGGGTAAAGGT and primer 2: GCAAGTCGGTGCTTACCTG (2)) or a gel analysis of restriction of the same DNA fragment by Msp A1I (New England Biolabs, Ipswich, MA).

### Statistical methods

Follow up was calculated from the time of diagnosis to date of last follow-up. The rate of death was estimated using Kaplan-Meier methods. Statistical analysis was performed using the SPSS^® ^statistical package (SPCC Inc., Chicago, IL), and JMP^® ^software (SAS Institute, Cary, NC). Assessment of the correlations between genetic carrier status and MDM2 was carried out using the Fisher Exact test or Chi-Squared test. A p value < 0.05 was considered significant. Similar methodology was used to assess the correlation between the frequency of other cancers and MDM2 SNP309 status

## Results

We evaluated DNA samples from 180 Ashkenazi breast cancer BRCA1/2 positive (Table 1), and from 272 patients who tested negative for these three Ashkenazi mutations.

**Figure 1:**
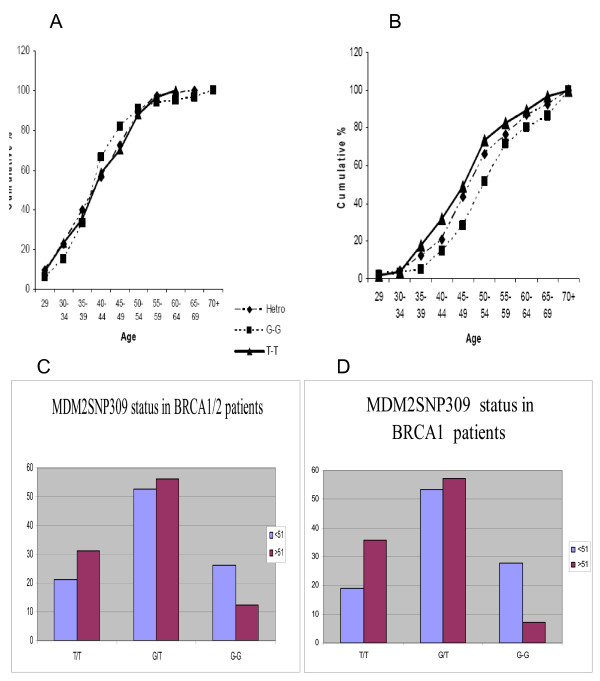
**Cancer onset age in the BRCA1/2 mutation carriers (A) and BRCA1/2 non carriers (B), according to their MDM2 SNP-309 subtypes**. **C **and **D **prevalence of the SNP MDM309 genotypes in women above and bellow 51 years old – C total carrier population and D only BRCA1 mutation carriers.

There was no significant difference between the prevalence of the different MDM2 SNP309 alleles between the BRCA1/2 patients (Table 2a) and the non carrier group (Table 2b). Disease onset age of breast cancer did seem to vary according to MDM2 309SNP genotype in the BRCA1/2 groups (Fig 1A). Similar to the data presented for women without high expression of estrogen receptors [6], there was a trend for disease onset at a higher age in the G/G MDM2 309 group than in the T/T group in the non-BRCA mutation carriers (Fig. 1B and Table 3). Our data did not distinguish between high and low estrogen receptor expressers therefore we could not asses a correlation between high estrogen receptor expression and disease onset age in a similar manner to that performed by Bond and colleagues.

**Table 2:** Prevalence of MDM2SNP309 subtypes

a: Prevalence of the MDM2 SNP309 subtypes in BRCA mutation carriers				
	**BRCA1**	**BRCA2**	**BRCA1+2**	**Total**

Homozygotes G-G	29(26.1)%	16(23%)		45(25%)
**Hetrozygotes**	55(49.5%)	39(57.3%)		94(52.2%)
Homozygotes T-T	27(24.3%)	13(19.1%)	1(100%)	41(22.7%)
**Total**	111	68	1	180

b: Prevalence of the MDM2 SNP309 subtypes in the overall population				

	***BRCA Carriers***		***Non Carriers***	
	Frequency	Percent	Frequency	Percent

Homozygotes G-G	45	25.00%	68	25.00%
Hetrozygotes	94	52.20%	139	51.10%
Homozygotes T-T	41	22.80%	65	23.90%
Total	180		272	

**Table 3:** Cancer onset age.

	Non carrier Mean age	Carrier Mean age
Hetro	52.6	42.3
T-T	49.1	43.9
G-G	55.2	40.4
P value	**0.005**	0.2

Breast cancer onset age in the BRCA1/2 carriers and non carrier group.

Recently Yarden and colleagues divided the breast cancer patient group to those with tumors diagnosed under the age of 51 and those above 51. In a similar patient group to ours they demonstrated a significantly decreased percentage of MDM2 309 G/G patients in the over 51 BRCA1/2 carriers compared to the other genotypes[4]. However when we have done similar analysis we did not note such a difference. When we limited our comparison to MDM2 309 G/G versus T/T (without taking into account the heterozygotes group) we noted in the BRCA1/2 group a trend for lower frequency of G/G in the over 51 year old group (p = 0.069) and this trend reached statistical significance in BRCA1 positive group only (Fig 1c and 1d p = 0.049). The number of patients over 51 was limited and these results which are of borderline statistical significance must be regarded as "hypothesis generating data" and be confirmed in a much larger data set. Thus it seems that at least in our cohort the effects of MDM2309 genotype on cancer onset age are very limited.

**Figure 2:**
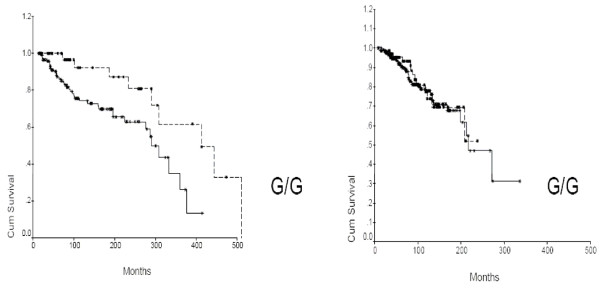
**Survival analysis of BRCA1/2 mutation carriers (A) and non carriers (B) according to their MDM2 SNP type**. SNP309 G/G carriers were compared to the combined group of SNP309 G/T and SNP309 T/T. In the BRCA carrier group there were 41 G/G patients of whom 7 died (median survival 412 months) while in the combined SNP309 G/T and T/T group there were 126 patients of whom 35 died (median survival 301 months). According to a log-rank test the significance of the difference in survival was P = 0.0086. In the control group the survival for the MDM2 SNP309 G/G carriers was was not statistically different (p = 0.8).

Our next step was to study the survival of the different subpopulations according to the three different genotypes (T/T, G/G or G/T at the -309 position of the MDM2 gene). There was a distinct survival advantage in the BRCA1/2 mutation carrier group for the patients homozygous for G/G at the -309 position, compared to the patients harboring the SNP309 G/T or T/T (Fig 2a p = 0.0086 log rank test, median survival 290 versus 412 months). This advantage was not found in the non BRCA1/2 carriers (Fig. 2b). Cox multivariate analysis did not reveal modification of the MDM2 SNP309 effect on survival due to cancer onset age.

## Discussion

In the current study we analyzed the relationship between MDM2 309 SNP status and cancer in a large group of Ashkenazi patients including a large group of BRCA1/2 mutation carriers Our results revealed high percentage of MDM2 309 SNP in this population of around a quarter of all women. This high prevalence of MDM2 309 SNP G/G in Ashkenazi women has been demonstrated by others before us. Yarden and colleagues recently presented data supporting the notion that the relative enrichment in MDM2 309 SNP G/G in Ashkenazi is a result of a selection process favoring this single nucleotide polymorphism[16]. Currently we don't understand the possible advantages of this polymorphism which has been linked to increased incidence of cancer, though recently in the 2008 AACR meeting A Levine presented preliminary evidence supporting a role for the p53 pathway(thus also MDM2) in regulation of newborn numbers.

We did not detect significant difference in cancer onset age in BRCA1/2 carriers harboring the different MDM2 SNP309 (Fig 1). These results are similar to those presented by Eccles and colleagues in a small study of BRCA1 positive patients [17]. Similarly Wasieleski and colleagues have not noted a higher frequency of MDM2 309 SNP G/G in younger BRCA1/2 breast cancer patients[5]. In contrast to that Yarden et al did find an earlier onset age for patients under 51 who harbor BRCA 1/2 mutations and MDM2 309 SNP G/G [4]. When we analyzed our data regarding onset age by dividing the whole patient group into those younger and older than 51, we found only in the BRCA1/2 carrier group a decreased percentage of MDM2 309 SNP G/G in the over 51 patient group (Fig 1c and 1d). However only when comparing this percentage to that of MDM2 309 SNP T/T in the BRCA1 carrier group (excluding the BRCA2 carriers) MDM2SNP G/G prevalence among the over 51 patients percentage was statistically smaller compared to the MDM2SNP T/T group (p = 0.049) (see fig 1c and 1d). Thus it seems while we detected some effect of MDM2SNP G/G on cancer prevalence in the younger than 51 years old, this effect was significantly smaller compared to that described by Yarden and colleagues[4].

The effect of the MDM2 SNP 309 G/G on cancer risk has been recently reviewed in a meta- analysis by Wilkeming and colleagues[18]. These authors concluded that "The data show that SNP309 alone has little or no effect on the risk of common cancers, but it might modify the time of tumor onset and prognosis". If one only studies the effects on median onset age of breast cancer in BRCA 1/2 patients the effect is small indeed, yet we also studied effects of MDM2 polymorphysm on survival in this group and here our results suggests a more profound role for the MDM2 SNP309 polymorphism.

When combining the BRCA1 and BRCA 2 mutation carriers we noted significantly better prognosis for the MDM2 SNP309 G/G carriers than for the other MDM2 SNP309 carriers (p = 0.0086). This are supposedly the tumors with higher levels of MDM2 expression. Cancers in BRCA1 and BRCA2 mutation carriers have substantial difference between them[19]. However in both BRCA1 and BRCA2 a special link to p53 has been described in animal and human. Furthermore, both BRCA1 and BRCA2 can form a complex with distinct cellular roles. A higher percentage of p53 mutations have been detected in the tumors of both BRCA1/2 mutation carriers. Thus it seems that similar mechanisms relating to these tumors and P53 (and therefore MDM2) might take place.

Since MDM2 is an oncogene it seems counterintuitive that patients in which higher levels of MDM2 are expected will have longer survival. A possible explanation for such a counterintuitive result is based on our understanding of the role of p53 in carcinogensis. The presence MDM2 SNP309 G/G may lead to the overexpression of MDM2 and therefore cause downregulation of p53 in an effective manner and render mutational inactivation of p53 unnecessary. Thus tumors with MDM2 SNP309 G/G might still harbor a potentially active p53. Indeed, when Allazzouzi and colleagues studied p53 in colon cancer of SNP309 G/G carriers), they found a higher tumor prevalence of non dominant p53 mutations[20]. When p53 is downregulated in the tumor not through mutational inactivation but through overexpression of an inhibitor certain treatments may allow reactivation of p53. Reactivating P53 may allow better response to anticancer agents. It is noteworthy that DNA damaging agents can significantly lower the levels of active MDM2 thus allowing the reactivation of p53 [21-23].

Therefore a possible scheme to explain a role for MDM2 SNP309 G/G in BRCA tumors could be that overexpression of MDM2 in BRCA1/2 tumors results in down-regulation of p53 and allows the development of BRCA1/2 tumors similar to the effect induced by a mutation in p53. Cells overexpressing MDM2 still contain p53 that in certain conditions. in which MDM2 is inactivated might be functional. As described MDM2 is inactivated by DNA damaging agents which includes chemotherapies. This hypothesis concurs with the hypothesis suggested by Mathoulin-Portie in their study of anthracyclin treated breast cancer women ([12]see introduction). These authors suggested that the better survival observed for p53+MDM2 + patients compared for the P53+MDM2- chemotherapy is a result of a different kind of p53 found in these tumors.

MDM2 SNP309G/G effects on BRCA1/2 positive tumors might be similar to that of the oncogene BCL6 on diffuse large cell lymphomas. Similarly to MDM2, BCL6 can down-regulate p53 function (though through transcriptional rather than post transcriptional control). This has been proposed as one of the main mechanisms of oncogenic transformation induced by this protein[24]. Moreover similarly to MDM2 it has been recently demonstrated that genotoxic stress can cause downregulation of BCL6[25]. Interestingly, BCL6 expression in diffuse large B cell lymphomas has been shown to strongly correlate with better survival in patients with this disease[26]. Thus, both BCL6 and MDM2 may cause down-regulation of wild type p53 and perhaps allow the emergence of tumors with non dominant negative p53. Such tumors might ultimately be more sensitive to therapy than tumors with mutated p53 if the anti-tumor therapy resulted in reactivation of a functional p53. When suggesting this hypothesis its important to note that both BCL6 and MDM2 have a variety of roles unrelated to p53 that could explain association between their increased expression and improved prognosis [1].

Recently two articles assessing the relationship between patients' survival and the MDM2 SNP309 were published. However, it is important to note that the patients in those studies suffered from a different disease than those in the current study and were treated with different therapies than those commonly used for treatments of breast cancer.

## Conclusion

Our data demonstrate that patients with BRCA1/2 breast cancers harboring any of the three MDM2 SNP309 had similar cancer onset ages. Only by studying the over 51 years old group of patients (presumably post menopausal) we could detect a difference between the different MDM2 SNP309 genotypes – only in the BRCA1 mutation carriers. The relative percentage of the MDM2SNP309G/G carriers was reduced in this group of patients compared to the younger than 51 years old (p = 0.049). While this results concurs with those of Yarden and colleagues they were obtained from a small number of patients and thus need reaffirmation in larger studies.

MDM2 SNP309G/G BRCA1/2 carriers had significantly longer survival compared to the combination of the other MDM2 SNP309 subgroups (p = 0.0086). This somewhat surprising result may be a result of differential sensitivity to adjuvant therapy in this subgroup. Further studies on MDM2 SNP309 may provide important insights as to factors effecting tumorigenesis and drug sensitivity in BRCA1/2 mutation carriers.

## Competing interests

The authors declare that they have no competing interests.

## Authors' contributions

Initiation of research HN, TP. Build up of database TP, TH, LK Collection of samples, DNA and statistical analysis – TH, SM. Discussion and Analysis of results HN, EP, TP, NS, LK.

## Pre-publication history

The pre-publication history for this paper can be accessed here:

http://www.biomedcentral.com/1471-2407/9/60/prepub
